# Statistical Investigation of High Culture Contamination Rates in Mycobacteriology Laboratory

**DOI:** 10.3389/fmicb.2022.789725

**Published:** 2022-05-06

**Authors:** Muatsim Ahmed Mohammed Adam, Rasha Sayed Mohammed Ebraheem, Shahinaz Ahmed Bedri

**Affiliations:** ^1^National Public Health Laboratory (NPHL), National Tuberculosis Reference Laboratory (NTRL), Khartoum, Sudan; ^2^National Public Health Laboratory (NPHL), DG, Khartoum, Sudan

**Keywords:** culture, contamination, management review, continuous improvement, laboratory diagnosis, tuberculosis, quality indicators

## Abstract

**Background:**

Culture of *Mycobacterium tuberculosis* remains the gold standard in mycobacteriology laboratories, constrained by the very high risk of contamination; therefore, contamination rate is an important key performance indicator (KPI) for laboratory monitoring and evaluation processes.

**Aim:**

This study aimed to investigate the factors that contribute to elevated contamination rates in the Sudan National Tuberculosis Reference Laboratory.

**Method:**

A laboratory-based retrospective study was applied; a TB culture register-book was carefully reviewed and data from 2 January 2019 to 31 December 2019 were entered, cleaned, and analyzed using IBM SPSS 20. A multivariate logistic regression model was performed to examine two dependent variables, the massive contamination, and the single tube contamination against predictors of reason for cultivation, type of specimen, experiment team, and the quarter of cultivation.

**Results:**

It has been found that in 2019 contamination rates were frequently higher; the highest rates were recorded in January and November, 28.2 and 25.2%, respectively. August is an exception with an accepted contamination rate of 4.6%. Of 1,149 specimens requested for culture, 945 (82.2%) samples were eligible to be included in multivariate logistic regression analysis. The team conducting the experiment was significantly associated with a high single tube contamination *p value* 0.007; adjusted odds ratio AOR 3.570 (1.415–9.005). The correlation between the single tube contamination and the massive contamination is significant; *p value* 0.01.

**Conclusion:**

The study concludes that high culture contamination is the greatest risk to the quality of laboratory service and can end in either the loss of specimens or delay in the decisions of initiating patient treatment. In addition, the low quality or incompleteness of data increases the uncertainty and undermines the measurement of key performance indicators.

## Introduction

Sudan is a moderately tuberculosis (TB) burdened country. In 2014 the prevalence was 159/100,000; it is higher among adults, in urban settings, and in the male population, although 11–13% of all notified cases are children. In 2017, the estimated TB incidence was 77/100,000, for a total of 31,000 cases and a mortality rate of 12/100,000. Comparatively, TB case notifications remained steady for the last decade in a range of (19,817–22,097). In 2016, the total notification was 21,091 cases. Among them, 19,305 (91.5%) were new, while 1,786 (8.5%) were retreatment cases. Of the new cases, 6,520 (30.9%) were smear-positive, 6,167 (29.2%) were smear-negative, 4,951 (23.5%) were extra-pulmonary, and in 1,667 (7.9%) sputum was not done. Last year there were three presumptive cases of extensively drug resistant tuberculosis (XDR-TB); only one of them was confirmed and the notified multi drug resistant tuberculosis (MDR-TB) cases were 220 (CNCDCD et al., 2018).

In Sudan, the GeneXpert network was designed and consists of three levels; it was managed and supervised centrally by the National Tuberculosis Reference Laboratory (NTRL), connected with 18 state laboratories located in capital cities, equipped with one machine or more. Based on MDR notifications, it was expanded to serve 72 sites in localities. An external quality assessment (EQA) system was planned but not yet implemented. The GeneXpert network was employed as the earliest diagnostic tool in the diagnostic algorithm which prioritizes screening and early identification of rifampicin resistant cases among TB suspect and MDR-TB high-risk groups, such as TB retreatment cases, MDR-TB contact, HIV patients, and prisoners. To scale up the programmatic management of drug resistant tuberculosis; sputum smear microscopy was only dedicated for the monitoring of treatment of first-line anti TB drugs (FLDs). So far, culture is the gold standard diagnostic method in the mycobacteriology laboratory.

Several types of culture systems have been developed, however, Löwenstein–Jensen medium (LJ) is considered the reference mycobacterial growth medium (Rageade et al., 2014). Solid egg-based cultures are cheaper and safer, more specific, and allow better visibility of colonial morphology compared to liquid systems (Kidenya et al., 2013; Ahmad et al., 2017). Cultures are more sensitive and significantly increase the number of notified cases (Asmar and Drancourt, 2015). However, they are time-consuming when compared to molecular techniques and sputum smear microscopy. Additionally, cultures provide the necessary isolates for conventional drug susceptibility testing (DST) to provide a definitive diagnosis, therefore, molecular techniques and sputum smear microscopy are not replacing the culture of *M. tuberculosis* (World Health Organization, 1998; Chihota et al., 2010). Cultivation of mycobacteria is the only method that enables differentiation between viable and dead bacterial cells. This characteristic was frequently recruited to monitor treatment response, to confirm patient sterility and sputum conversion, as well as to ensure the breaking of transmission cycles and to document cure of patients (Su et al., 2011; World Health Organization, 2014; Lawn and Nicol, 2015; Wong et al., 2016).

The accuracy of technical manipulation of analytical and pre-analytical processes either negatively or positively impacts the prone of mycobacterial culture contamination. Soft decontamination procedures due to the constraints of less time or lower concentration might increase the possibility of contamination occurrence. In addition, poor quality of specimens, delay in transportation to the laboratory, as well as poor quality of culture media and incubation conditions might be addressed as possible causes of contamination (World Health Organization, 2011). In this study, we aimed to explore other factors that might contribute to raised contamination rates in the Sudan National Tuberculosis Reference Laboratory.

## Materials and Methods

### Study Setting

Sudan National Tuberculosis Reference Laboratory (NTRL) is the sole reference TB laboratory in the country; it stands at the top of the hierarchical network of four regional TB culture laboratories, 18 smear microscopy quality assurance (EQA) laboratories, and 327 tuberculosis management units (TBMUs). The diagnostic menu includes a variety of conventional laboratory tests: Ziehl–Neelsen (ZN) and Phenolic-Auramine fluorescence microscopy (FM), egg-based cultures, first line, and second-line drug susceptibility testing (DST). In addition, the WHO endorsed molecular techniques Xpert^®^ MTB/RIF assay (Cepheid Inc., Sunnyvale, CA, United States) and Line Probe Assay GenoType MTBDR*plus* for rifampicin and isoniazid resistance (Hain Lifescience GmbH, Nehren, Germany), and GenoType MTBDR*sl* for XDR detection.

### Laboratory Culture

Sudan National Tuberculosis Reference Laboratory is adopting the Petroff method for sputum decontamination and homogenization. Standard operating procedure (SOP) describes the addition of an equal volume of 4% sodium hydroxide solution (4% NaOH) to the sputum sample, gentle shaking, and homogenization for 15 min. Neutralization of alkaline solution includes three without-centrifugation options: the use of hydrochloric acid (HCl) with phenol red indicator, use of phosphate buffer saline (PBS) pH 6.8, or direct inoculation onto two tubes of Ogawa acidified medium. Löwenstein-Jensen medium (LJ) Slants were incubated at 37°C for 8 weeks and inspected regularly for the recording of negative results. Culture contamination and the grades of positive results (scanty, +, ++, +++) were recorded immediately when visible mycobacterial growth appear.

### Data Collection and Data Management

The culture laboratory register book was carefully reviewed. It includes data about specimens, patients, and results of smear and culture. Data were categorized, double entered onto statistical package for social sciences (IBM SPSS statistics 20), checked, and cleaned. Category “Not recorded” was elaborated for not filled data from the original institution that requested the culture, unknown data, or data not transcribed from the request form inside the reference laboratory. Specimens that were requested and not received or not processed were excluded. Furthermore, information about sample collection, sample reception, and sample processing dates were not completely filled in the culture register book; therefore, these variables were also excluded from this study. Dried culture tubes indicate ignorance of early inspection of culture slants, undermines the good microbiological technique (GMT), and also ends to the loss of specimen; therefore, dried tubes were considered as contaminated. In case of two different direct and indirect smears positive results from the same specimen, or any other difference between positive results of each single culture tube, the highest grade was considered. For example, if the result was (++) and the result of the other tube was (+) the result of culture was recorded as (++).

### Statistical Analysis

In this study, the proposed predictors include the reason for cultivation, type of specimen, the team conducting the experiment, and the quarter of cultivation which referred to three successive months in the year (January to March, April to June, July to September, and October to December); each predictor that had complete data was illegible for statistical analysis. These predictors were examined separately against two dependent variables: the massive contamination (contamination of all three cultivated tubes) and contamination of single tube (contamination of only one or two cultivated tubes). A multivariate logistic regression model was applied to examine statistical associations at 95% confidence intervals, the reference category is first ordered ascending. Odds ratios, upper bound, lower bound, and *p* values were calculated, and a correlation between both types of contamination was determined.

### Ethical Consideration and Publication Approval

This study is based on secondary data, and it did not involve working with any type of human subjects, clinical specimens or interventions, patients’ interviews, or clinical trials; however, the proposal has been scientifically and ethically approved by the national public health laboratory ethical committee. The publication permission and approval were obtained from the general directorate of the National Public Health Laboratory (NPHL).

## Results

### Study Subjects and Study Population

**FIGURE 1:**
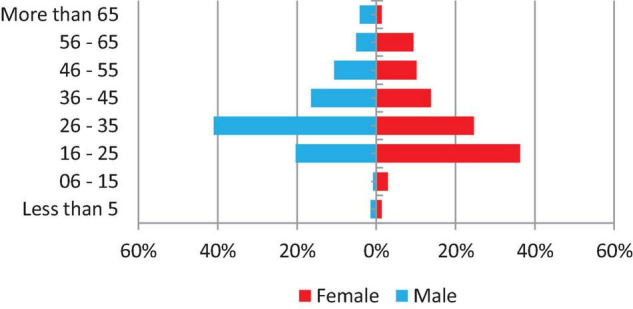
Demographic of TB presumptive and follow-up cases.

From 2 January 2019 to 31 December 2019, a number of 1,149 specimens were requested for culture in the National Tuberculosis Reference Laboratory, of which 1,125 (97.9%) were received and processed and have had a laboratory culture result. The majority of specimens (881–78.3%) were requested for treatment monitoring, whereas 83 (7.4%) were baseline specimens and the reason for culture request of 161 (14.3%) were not recorded. In addition, 1,040 (92.4%) specimens were sputum, 30 (2.7%) were other than sputum, and 55 (4.9%) were not recorded. Experiments were conducted by five principal operators in six work teams and the range of workload for culture is 51–159 specimens per month. Only 496 (43.2%) of patients’ demographic information is complete and shown in Figure 1.

### Laboratory Culture

After following the exclusion criteria, of the 1,125 registered samples, a total of 945 (84%) samples were eligible to be included in statistical analysis; of them, 656 (73%) were smear-negative, 255 (16.7%) were smear-positive, and the remaining 97 (10.3%) were not recorded. A breakdown of results is shown in Table 1.

**TABLE 1:** Frequency of smear microscopy result, reason of specimen cultivation, and culture result.

Reason for culture			Culture						Total
			Negative	Scanty	1+	2+	3+	Contamination	
Base line	Smear	Negative	28	2	3	0	0	1	34
		1+	4	1	9	6	1	0	21
		2+	1	0	4	1	4	0	10
		3+	0	0	0	1	2	0	3
		Not recorded	4	0	4	1	0	1	10
	Total		37	3	20	9	7	2	78
Follow-up	Smear	Negative	624	8	10	1	0	13	656
		Scanty	10	6	0	0	0	0	16
		1+	57	3	18	7	4	1	90
		2+	2	1	5	3	2	0	13
		3+	2	0	1	2	0	0	5
		Not recorded	73	3	3	0	0	8	87
	Total		768	21	37	13	6	22	867
Total	Smear	Negative	652	10	13	1	0	14	690
		Scanty	10	6	0	0	0	0	16
		1+	61	4	27	13	5	1	111
		2+	3	1	9	4	6	0	23
		3+	2	0	1	3	2	0	8
		Not recorded	77	3	7	1	0	9	97
	Total		805	24	57	22	13	24	945

*The majority of the specimens (91.7%) were requested for treatment monitoring reasons while the remaining (8.3%) were requested for diagnosis reasons. One half of the results of smear microscopy for the diagnostic specimens were positive and the other half was negative. Noticeably, (13%) of the data were missing. Of the follow up, (75%) of specimens were smear negative, (10%) of data were missing, and (15%) were smear positive. When all specimens were pooled, 690 of them (73%) were smear negative, 97 (10.3%) of the data were not recorded, and the rest (17%) were smear positive.*

**TABLE 2:** Correlation between single tube contamination and massive contamination.

		Single tube contamination	Massive contamination
Single tube contamination	Pearson Correlation	1	0.262**
	*P value*		0.000
	N	945	945
Massive contamination	Pearson Correlation	0.262**	1
	*P value*	0.000	
	N	945	945

The computed 2 × 2 table revealed a significant weak correlation between massive contaminations, and single tube contamination at 0.01; correlation coefficient was 0.262. Details are shown in this table. **Correlation is significant at the 0.01 level.

### Culture Contamination

**FIGURE 2:**
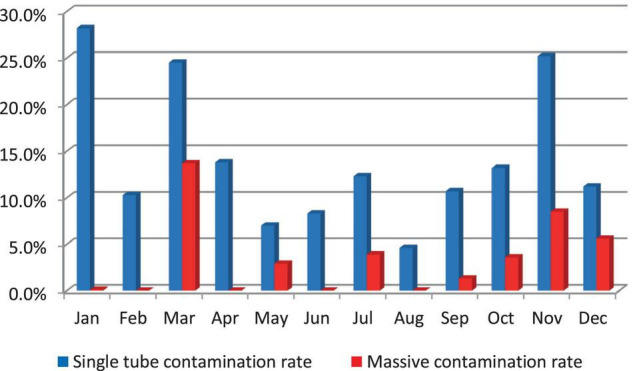
Culture contamination rate, 2019.

Massive contamination rates were higher in March (13.7%) and in November (8.5%). They decreased to 0.1% in January and did not occur (0%) in February, April, June, and August; Single tube contamination rates were higher in January (28.2%), November (25.2%), and in March (24.5%) (Figure 2).

### Predictors of High Culture Contamination Rates

Out of 1,125 specimens included in this study, 180 (16%) samples were excluded from the statistical analysis. The remaining 945 samples (84%) had complete data regarding independent variables and are eligible to be included in the statistical analysis; details were shown in Tables 3, 4.

**TABLE 3:** Multivariate logistic regression of single tube contamination.

Predictor	Category	*p* value	AOR*	95% Confidence interval	
				Lower bound	Upper bound
Reason of culture	Base line	0.378	1.289	0.733	2.268
	Follow-up		1		
Type of specimen	Sputum	0.678	0.781	0.242	2.516
	Other		1		
Quarter of cultivation	Q1^a^	0.060	1.671	0.978	2.853
	Q2^b^	0.661	0.883	0.507	1.538
	Q3^c^	0.103	0.650	0.388	1.091
	Q4^d^		1		
Team of work	A	0.492	1.406	0.532	3.718
	B	0.120	2.168	0.817	5.751
	C	0.077	2.373	0.910	6.189
	D	0.289	1.697	0.639	4.509
	E	0.007	3.570	1.415	9.005
	F		1		.

^a^January to March, ^b^April to June, ^c^July to September, ^d^October to December. Multivariate logistic regression reveals a significant association between experiment team and single tube high contamination rate p value 0.007; AOR 3.570 (1.415–9.005). * Adjusted odds ratio.

**TABLE 4:** Multivariate logistic regression of massive culture contamination.

Predictor	Category	*p* value	AOR*	95% Confidence interval	
				Lower bound	Upper bound
Reason of culture	Base line	0.778	0.778	0.135	4.468
	Follow-up		1		
Type of specimen	Sputum	0.075	0.093	0.007	1.273
	Other		1		
Quarter of cultivation	Q1^a^	0.446	1.579	0.488	5.112
	Q2^b^	0.097	0.230	0.041	1.303
	Q3^c^	0.079	0.274	0.065	1.163
	Q4^d^		1		
Team of work	A	0.200	0.140	0.007	2.830
	B	0.136	0.102	0.005	2.056
	C	0.879	0.832	0.078	8.919
	D	0.167	0.149	0.010	2.215
	E	0.622	1.758	0.187	16.537
	F		1		

^a^January to March, ^b^April to June, ^c^July to September, ^d^October to December. Multivariate logistic regression reveals there is no significant statistical associations between massive contamination and proposed predictors. * Adjusted odds ratio.

## Discussion

The WHO laboratory quality management system LQMS document recommends management reviews of all information gathered in the laboratory records regularly (World Health Organization, 2011); the typical period for conducting a management review is annually to provide useful information about areas for improvement (International Organization for Standardization, 2005, 2012).

The accepted culture contamination rate in Löwenstein–Jensen (LJ) solid medium is 3–5% (European Centre for Disease Prevention Control, 2016). In this report culture contamination rates were higher, up to 28.2%. Inaccuracy of analytical processes are the most likely proposed cause of high contamination rates; however, several other factors are thought to contribute to increasing the contamination rate. High contamination rate in solid egg-based culture media is frequently seen in different settings, such as Kenya (Okumu et al., 2017) and India (Sharma et al., 2017). Further, as in other low-income countries, the laboratories infrastructure was fragile, and this could contribute to additional causes of high contamination rates (Engel et al., 2016). In this study, the team conducting the experiment was significantly associated with single tube high contamination rate; similar findings were perceived in other reports (Reddy et al., 2014). Sudan National Tuberculosis Reference Laboratory was fully staffed with experienced qualified technical personnel; however, this association might be attributed to the uneven level of proficiency or other socioeconomic factors.

Major limitations of this study were due to the missing, not recorded, or not transcribed data; this could increase the uncertainty of generated information rather than the appropriate interpretation of the study findings (Dilraj et al., 2015), as in this study approximately only one-half of data regarding age and gender was completed. However, TB epidemiology of the laboratory attendees was similar to international status (Dodd et al., 2016; Marcoa et al., 2018). Secondly, it could decrease the ability of extracting and assessing other key performance indicators in the TB culture laboratory which could be measured routinely. These indicators were highlighted in several studies. Cultures can increase TB detection by 30–50% (Demers et al., 2012; Kassaza et al., 2014). In this study, due to the negative impact of not recorded data, it was not possible to measure the recovery rate indicator. Other indicators, such as turnaround time, was also reported to affect the quality of the laboratory service (Stapleton and Sammond, 2012). The effect of delay between the collection, dispatching, and processing was seen in Burkina Faso (Kaboré et al., 2014; Reddy et al., 2014). To date, Sudan National Tuberculosis Reference Laboratory relies upon a paper-based recording system, which increases the chances of error. These limitations can partially be fixed by the development of a Laboratory information management system (LIMS); it is an important tool for the management of laboratory data on samples, instruments, results, and quality indicators. The LIMS can also support laboratory performance to acquire accreditation (Parsons et al., 2011).

## Conclusion

The study concludes that high culture contamination is the greatest risk to the quality of laboratory service and can end in either the loss of specimens or delay in the decisions of initiating patients’ treatment. In addition, the low quality of data increases the uncertainty and undermines the measurement of key performance indicators. Furthermore, the team conducting the experiment was found to be associated with high culture contamination rate. Therefore, the study recommends that all culture laboratory staff should be on the same level of knowledge and proficiency according to their role and should be under regular restricted supervision; in addition, laboratory management should have to use regular assessment tools for internal audits.

## Data Availability Statement

The original contributions presented in the study are included in the article/supplementary material, further inquiries can be directed to the corresponding author.

## Author Contributions

MM wrote the preliminary draft. RE contributed to data entry and data cleaning. SB critically reviewed the manuscript. All authors contributed to the article and approved the submitted version.

## Conflict of Interest

The authors declare that the research was conducted in the absence of any commercial or financial relationships that could be construed as a potential conflict of interest.

## Publisher’s Note

All claims expressed in this article are solely those of the authors and do not necessarily represent those of their affiliated organizations, or those of the publisher, the editors and the reviewers. Any product that may be evaluated in this article, or claim that may be made by its manufacturer, is not guaranteed or endorsed by the publisher.
